# Downregulation of miR-199b is associated with distant metastasis in colorectal cancer via activation of SIRT1 and inhibition of CREB/KISS1 signaling

**DOI:** 10.18632/oncotarget.9042

**Published:** 2016-04-27

**Authors:** Zhan-long Shen, Bo Wang, Ke-wei Jiang, Chun-xiang Ye, Cheng Cheng, Yi-chao Yan, Ji-zhun Zhang, Yang Yang, Zhi-dong Gao, Ying-jiang Ye, Shan Wang

**Affiliations:** ^1^ Department of Gastroenterological Surgery, Peking University People's Hospital, Beijing, 100044, PR China; ^2^ Laboratory of Surgical Oncology, Peking University People's Hospital, Beijing, 100044, PR China; ^3^ Department of General Surgery, Tangshan Gongren Hospital, Hebei Medical University, Tangshan, Hebei, 063000, PR China

**Keywords:** miR-199b, SIRT1, KISS1, colorectal cancer, metastasis

## Abstract

The progression of distant metastasis cascade is a multistep and complicated process, frequently leading to a poor prognosis in cancer patients. Recently, growing evidence has indicated that deregulation of microRNAs (miRNAs) contributes to tumorigenesis and tumor progression in colorectal cancer (CRC). In the present study, by comparing the miRNA expression profiles of CRC tissues and corresponding hepatic metastasis tissues, we established the downregulation of miR-199b in CRC metastasis tissues. The decrease in miR-199b expression was significantly correlated to late TNM stage and distant metastasis. Moreover, Kaplan–Meier curves showed that CRC patients with high expression level of miR-199b had a longer median survival. Functional assays results indicated that the restoration of miR-199b considerably reduced cell invasion and migration *in vitro* and *in vivo*, and increased the sensitivity to 5-FU and oxaliplatin. Further dual-luciferase reporter gene assays revealed that SIRT1 was the direct target of miR-199b in CRC. The expression of miR-199b was inversely correlated with SIRT1 in CRC specimens. SIRT1 knockdown produced effects on biological behavior that were similar to those of miR-199b overexpression. Furthermore, through Human Tumor Metastasis PCR Array we discovered KISS1 was one of the downstream targets of SIRT1. Silencing of SIRT1 upregulated KISS1 expression by enhancing the acetylation of the transcription factor CREB. The latter was further activated via binding to the promoter of KISS1 to induce transcription. Thus, we concluded that miR-199b regulates SIRT1/CREB/KISS1 signaling pathway and might serve as a prognosis marker or a novel therapeutic target for patients with CRC.

## INTRODUCTION

Colorectal cancer (CRC) is the third most common cancer in the world, with nearly 1.4 million new cases diagnosed in 2012 [1]. Rather than primary tumors, distant metastasis, especially liver metastasis, the current therapy for which is largely unsuccessful, is the leading cause of death in patients with CRC [2, 3]. Therefore, a better understanding of the metastatic process and the molecular mechanism underlying liver metastasis is critical to the improvement of the development of effective diagnostic and therapeutic strategies for patients with CRC metastasis.

In recent years, posttranscriptional regulation has been demonstrated as a key process involved in cancer metastasis [4]. In particular, microRNAs (miRNAs), small non-coding RNAs post-transcriptionally regulating gene expression, have been reported to act as a suppressor or promoter in the progression of metastasis in diverse cancer types [5–7]. It has been suggested that miR-199b plays a central role in cancer progression and metastasis, including breast cancer [8], prostate cancer [9], osteosarcoma [10], and medulloblastoma [11]. In spite of the accumulating evidence highlighting the importance of miR-199b in cancers, to the best of our knowledge, no study has investigated the role of miR-199b in CRC progression and metastasis.

Sirtuin 1 (SIRT1) is one of seven members of the sirtuin family of nicotinamide adenine dinucleotide (NAD+)-dependent class III histone deacetylases [12, 13]. The activity of SIRT1 is essential for the acetylation of histones and non-histones which are involved in the regulation of cell metabolism [14], differentiation [15], proliferation [16], and invasion [17]. SIRT1 overexpression stimulates cells invasion and metastasis by enhancing the expression of matrix metalloproteinase (MMPs) while suppressing that of E-cadherin in various caner types [5, 18, 19]. However, little is known about the function of SIRT1, and it has not yet been fully expounded in the progression of CRC metastasis.

KISS1 is a member of the still-expanding family of metastasis suppressors, which are defined by their ability to block metastasis without preventing primary tumor development [20]. Previous studies have shown that KISS1 was closely associated with the prognosis of cancer patients, and the decreased expression of KISS1 results in increased metastatic potential and aggressive tumor progression [21, 22].

In the present study, we sought to identify SIRT1 as a target of miR-199b and explore the effects of miR-199b/SIRT1/KISS1 signaling in the progress of CRC metastasis. Our findings clearly evidenced a novel and negative role of miR-199b in the regulation of SIRT1, leading to the activation of CREB/KISS1 in CRC cells. Futhermore, miR-199b caused a suppressive effect on metastasis progression in these cells. In addition, miR-199b appears to be an effective prognosis predictor in patients with CRC.

## RESULTS

### Screening of differentially expressed miRNAs in CRC primary tissues and liver-metastasis tissues

**Figure 1:**
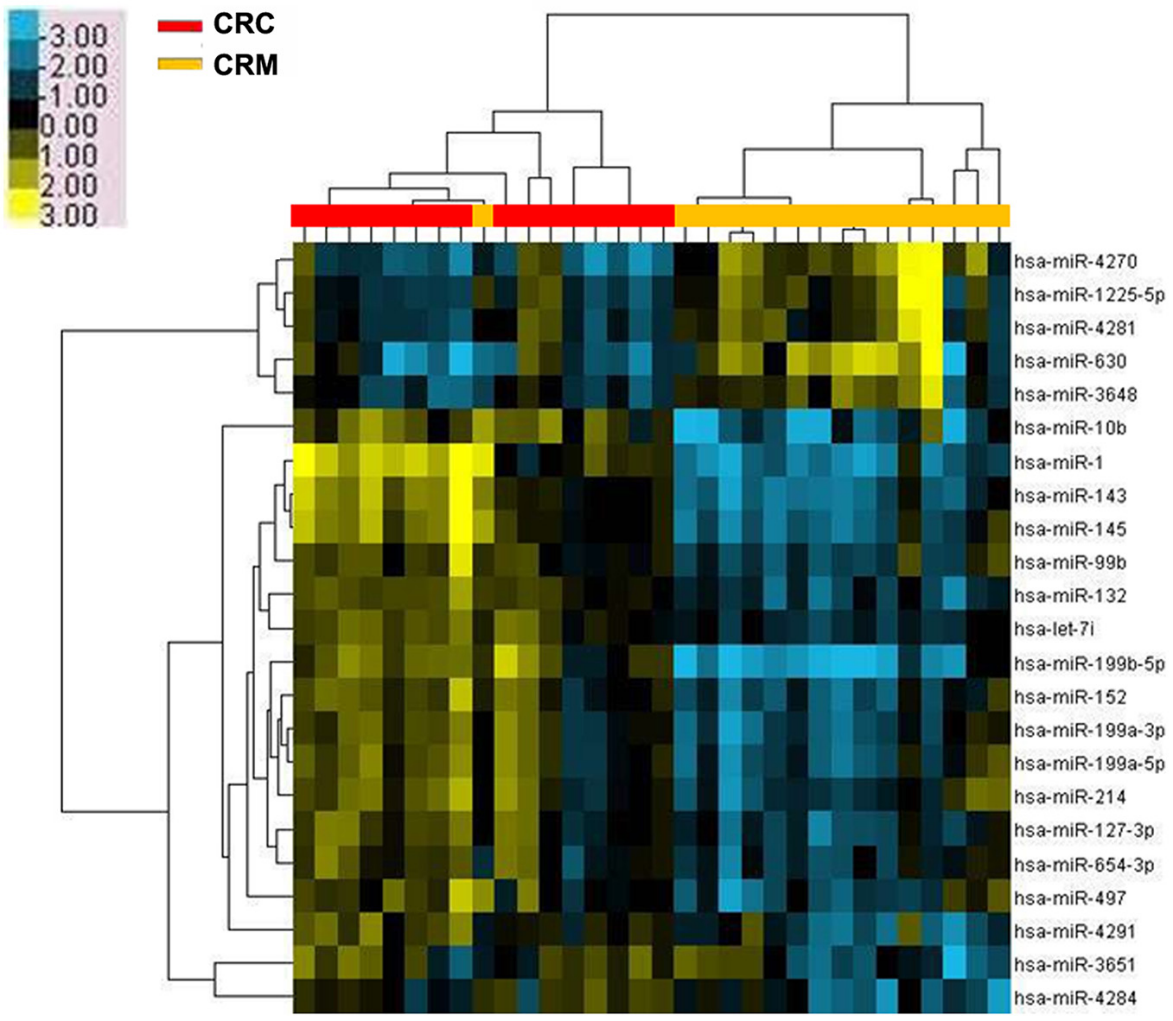
Microarray assay of miRNA expression in colorectal cancer tissues Hierarchical clustering of the expression values for mature miRNAs of CRC versus CRM tissues. Yellow indicates high relative expression, and blue indicates low relative expression. CRC: colorectal cancer; CRM: colorectal cancer metastases.

The comparison between the data obtained by miRNA microarrays of CRC primary tissues and liver-metastasis ones (Figure 1) showed a 2.0-fold change with a significant difference of a total of 5 upregulated miRNAs and 18 downregulated miRNAs identified in the tissue samples of liver metastasis (Table 1, *P*<0.05). Of these miRNAs, miR-4270 exhibited the highest degree of upregulation, whereas miR-199b expression manifested the most considerable decrease. In this study, we focused on elucidating the role of miR-199b in CRC progression.

**Table 1:** Differentially expressed miRNAs in colorectal liver metastases (CRM) compared with corresponding colorectal cancer primary tissues (CRC)

MiRNA	Fold change	Expressed in CRM	*P* value
hsa-miR-4270	4.4124584	up	0.001024669
hsa-miR-630	3.3995376	up	0.009981853
hsa-miR-1225-5p	2.4253035	up	0.004055073
hsa-miR-4281	2.1374993	up	0.013107119
hsa-miR-3648	2.1199193	up	0.009420886
**hsa-miR-199b-5p**	8.542169	down	3.20E-04
hsa-miR-1	8.012649	down	0.001132623
hsa-miR-143	5.208039	down	0.001024669
hsa-miR-10b	4.3175526	down	0.003524723
hsa-miR-145	4.1469736	down	0.002526151
hsa-miR-152	2.8310778	down	0.001024669
hsa-miR-132	2.807202	down	4.45E-04
hsa-miR-199a-3p	2.798662	down	6.37E-04
hsa-miR-199a-5p	2.5843542	down	0.001063863
hsa-miR-4291	2.5543244	down	0.001024669
hsa-miR-127-3p	2.4703388	down	6.37E-04
hsa-let-7i	2.2781188	down	3.20E-04
hsa-miR-99b	2.2129295	down	0.001583584
hsa-miR-214	2.1945717	down	0.002913084
hsa-miR-3651	2.0946753	down	0.009233502
hsa-miR-497	2.0822763	down	0.034518708
hsa-miR-4284	2.074375	down	0.026000313
hsa-miR-654-3p	2.0253823	down	0.002526151

### Verification of the decreased expression of miR-199b in CRC liver metastatic tissues

**Figure 2:**
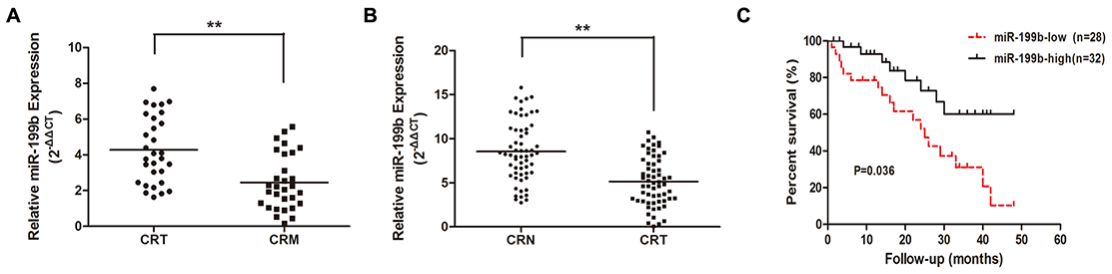
The expression level of miR-199b in tissue samples and its relevance to prognosis **A.** Relative expression of miR-199b in 30 pairs of CRC tissues and CRM tissues measured by qRT-PCR. **B.** Relative miR-199b expression level in CRC tissues and CRN tissues (n=60). **C.** Kaplan-Meier survival curve for overall survival assay by miR-199b expression in 60 CRC patients. P value was obtained by a log-rank test. CRT: colorectal cancer tissues; CRN: colorectal normal tissues. *P<0.05, **P<0.01

To validate the microarray data, we used qRT-PCR to evaluate the miR-199b expression level in another 30 paired samples of primary CRC tissues and such of liver metastasis. The results indicated that miR-199b was significantly downregulated in the liver metastatic tissues (Figure 2A, *P* <0.01), which exhibited a good consistency with the results of the microarray assay.

### Decreased miR-199b indicated poor prognosis of CRC patients

To assess whether miR-199b expression level was associated with the clinicopathological characteristics and prognosis of CRC patients, we determined miR-199b expression level in another 60 cases of CRC primary tissues and their corresponding adjacent normal tissues. The results revealed that the decreased miR-199b expression level in tumor tissues (Figure 2B) predicted a higher occurrence rate of late TNM stage (*P*=0.038) and distant metastasis (*P*=0.044), whereas no significant association with age, gender, tumor size, tumor differentiation, or lymph node metastasis (Table 2) was found. Meanwhile, the lower miR-199b expression level indicated a poorer prognosis in CRC patients who displayed a shorter median survival (Figure 2C). Furthermore, multivariate analysis confirmed that, together with distant metastasis, miR-199b was an independent prognostic factor for CRC (Table 3). The results above strongly indicated that downregulated miR-199b predicted poor prognosis in patients of CRC.

**Table 2:** The relationship between miR-199b expression and clinicopathologic characteristics in CRC patients

Parameters	miR-199b expression			Chi-square value	*P* value
	High (n=32)	Low (n=28)	Total (n=60)		
Age(y)					
≤60	13	12	25	0.031	0.861
> 60	19	16	35		
Gender					
Female	16	17	33	0.693	0.405
Male	16	11	27		
Tumor size(cm)					
≤2	10	14	24	2.188	0.139
>2	22	14	36		
Tumor differentiation					
Well/moderate	17	20	37	2.116	0.146
Poor	15	8	23		
TNM stage					
I+II	20	10	30	4.286	0.038*
III+IV	12	18	30		
Lymph node metastasis					
Positive	13	13	26	0.205	0.651
Negative	19	15	34		
Distant metastasis					
Positive	10	16	26	4.077	0.044*
Negative	22	12	34		

* Statistically significant (P<0.05)

**Table 3:** Multivariate analysis of factors related to overall survival in CRC patients

Variable	Multivariate analysis	
	HR (95%CI)	*P* value
Age	0.995 (0.961-1.029)	0.752
Gender	1.089 (0.380-3.122)	0.874
Tumor size	1.280 (0.500-3.276)	0.607
Tumor differentiation	2.125 (0.707-6.388)	0.180
TNM stage	0.751 (0.113-5.013)	0.768
Lymph node metastasis	1.199 (0.370-3.882)	0.762
Distant metastasis	7.378 (1.692-32.170)	0.008*
miR-199b	0.337 (0.136-0.833)	0.019*

HR, hazard ratio; CI, confidence interval;

* Statistically significant (P<0.05)

### MiR-199b suppressed CRC cell invasion and migration both *in vitro* and *in vivo*

**Figure 3:**
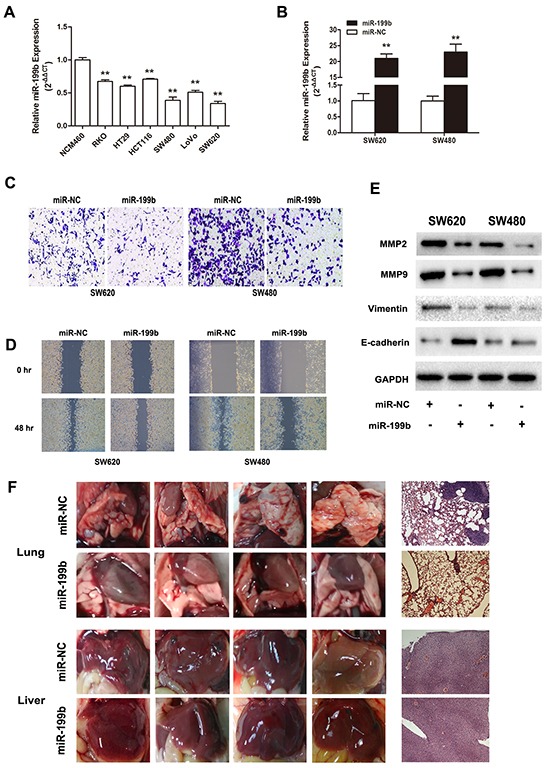
Metastasis suppressive effects of miR-199b in CRC cell lines *in vitro* and *in vivo* **A.** Relative miR-199b expression level in six CRC cell lines compared to the normal colorectal cell line NCM460. The average gene expression from NCM460 was designated as 1. **B.** Relative expression of miR-199b after transfected with miR-199b mimics and its negative control (NC), detected by qRT-PCR. The average miRNA expression from NC was designated as 1. **C.** The invasive ability of SW480 and SW620 cells was assessed by Transwell assay after overexpression of miR-199b. Statistics analysis was performed by counting the stained cells that invaded to the lower chamber under a light microscopy. **D.** Wound healing assay was applied to measure the migration ability of SW620 and SW480 cells. Quantification was performed by measuring the smallest clearance distance of the wound. **E.** Western blot analysis showed the expression levels of invasion related molecules MMP2 and MMP9, the epithelial-mesenchymal transition (EMT) marker E-cadherin and Vimentin after overexpression of miR-199b. **F.** Pictures of the lungs and livers in nude mice and their respective representative images of the tissues by hematoxylin-eosin (HE) staining. Metastases in lungs were observed in NC group but little in miR-199b group. No obvious metastasis formation could be seen in liver in these two groups. *P<0.05, **P<0.01.

To investigate the role of miR-199b in CRC metastasis, we transfected miR-199b mimics into SW480 and SW620 cells, both of which manifested the lowest miR-199b expression of the six colon cell lines in our laboratory (Figure 3A). Transwell and healing wound assays indicated that the upregulation of miR-199b resulted in a weaker ability of invasion and migration in both SW480 and SW620 cells (Figure 3C, D). In addition, after the overexpression of miR-199b, SW480 and SW620 cells showed a dramatic mesenchymal-epithelial transition (MET)-like transformation with significant upregulation of E-cadherin, as well as downregulation of Vimentin, MMP-2, and MMP9 (Figure 3E). In a nude mice model, the transfection with miR-199b lentivirus consistently repressed the metastasis of tumor cells in lungs and livers (Figure 3F and Supplementary Figure S1). These findings strongly support the implication that miR-199b exerts suppressive effect in CRC metastasis.

### MiR-199b enhanced the chemosensitivity of CRC cells to 5-FU and oxaliplatin

**Figure 4:**
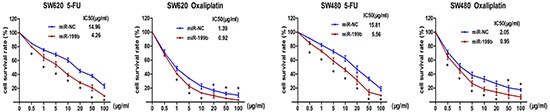
Chemosensitivity assay Chemosensitivity was evaluated after transfection with miR-199b by 5-FU and Oxaliplatin. Different concentrations were applied to the cells for 48 hours and cell viability was measured by CCK8 assay.

To explore the role of miR-199b in chemotherapy, we treated SW480 and SW620 cells with different concentrations of 5-FU and oxaliplatin, the two leading chemotherapeutic drugs used for clinical treatment of CRC. After transfection with miR-199b mimics, the IC_50_ of SW480 cells were significantly decreased both for 5-FU (5.56 vs. 15.81 μg/mL, *P*<0.01) and oxaliplatin (0.95 vs. 2.05 μg/mL, *P*<0.01; Figure 4). The miR-199b-transfected-SW620 cells displayed a similar result of IC_50_ changes (4.26 vs. 14.96 μg/mL for 5-FU and 0.92 vs. 1.39 μg/mL for oxaliplatin, *P*<0.01; Figure 4).

### SIRT1 is a direct target of miR-199b

**Figure 5:**
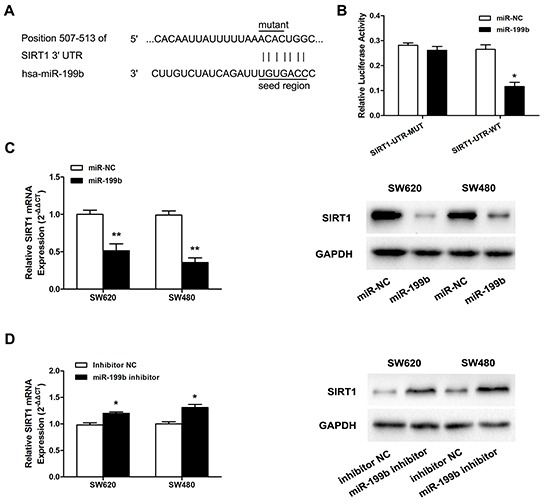
MiR-199b directly targets SIRT1 in SW620 cells **A.** Illustration of SIRT1 3′UTR as well as the seed sequence of miR-199b showing the predicted target region on the 3′UTR of SIRT1 mRNA. **B.** Dual-luciferase reporter assay with cotransfection SIRT1 3′UTR plasmids and miR-199b mimics. The relative luciferase activity was obtained by Firefly luciferase activity normalized against Renilla luciferase activity. **C.** The effects of overexpression of miR-199b on SIRT1 expression at mRNA level and protein level. **D.** The effects of inhibition of miR-199b on SIRT1 expression at mRNA level and protein level. *P<0.05, **P<0.01.

Since our previous studies showed that SIRT1 was significantly overexpressed in CRC [23], and the results obtained by the application of the online prediction software TargetScan 6.2, DIANA LAB, and PicTar all indicated the presence of miRNA responsive elements within the 3′UTR of SIRT1 as putative miR-199b targets (Figure 5A), we further evaluated whether SIRT1 was the direct target of miR-199b.

First, pmiR-RB-ReportTM-WT-SIRT1 and pmiR-RB-ReportTM–MUT-SIRT1 were co-transfected with miR-199b mimics into SW620 cells. As shown in Figure 5B, the relative luciferase activity in the reporter with wild type was significantly attenuated, whereas no obvious effect was observed in the mutant type 3′UTR of SIRT1. We further evaluated the expression level of SIRT1 mRNA and protein after the regulation of miR-199b expression. Obviously, decreased expression of SIRT1 was detected when miR-199b was overexpressed, whereas the inhibition of miR-199b led to upregulation of SIRT1 at both the mRNA and protein level (Figure 5C, D).

### Knockdown of SIRT1 leads to upregulation of KISS1 expression

Having identified that miR-199b controlled CRC invasiveness through SIRT1, we tried to continue investigating its downstream signaling pathways using the Human Tumor Metastasis PCR Array. The expression levels of all these genes are summarized in Supplementary Table S3 by comparing SIRT1-siRNA–transfected SW620 cells with NC-transfected cells. Among the changed genes KISS1 manifested the most elevated expression, whereas CDH6 displayed the most substantial degree of downregulation. KISS1 was recognized as a tumor metastasis suppressor and aroused our interest. We supposed that KISS1 was one of the critical downstream genes regulated by SIRT1. To confirm the data of the PCR array, we performed Western Blot in SW620 cells. As shown in Figure 7B, KISS1 was considerably upregulated after the treatment with SIRT1 siRNA.

### Association of SIRT1 expression level with miR-199b expression in CRC tissue samples

**Figure 6:**
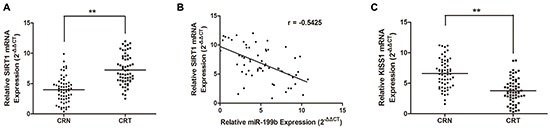
SIRT1 is upregulated while KISS1 is downregulated in CRC tissues and SIRT1 is antagonistically associated miR-199b expression **A.** Upregulation of SIRT1 was observed in CRC tissue samples compared with that in adjacent CRN ones by qRT-PCR. **B.** Spearman correlation was performed to assess relationship between expression levels of SIRT1 and miR-199b (r=−0.5425). **C.** KISS1 expression was decreased in CRC tissues compared to that in CRN ones. **P<0.01.

We analyzed the correlation of miR-199b and SIRT1 expression in CRC tissue samples. qRT-PCR assay showed that the expression of SIRT1 was much higher in CRC tissues than in noncancerous tissues (Figure 6A), while the KISS1 expression was lower (Figure 6C). Spearman's correlation showed that the expression of miR-199b was inversely related to that of SIRT1 in tissues (Figure 6B).

### The transcription factor CREB facilitates KISS1 expression through promoter activation

**Figure 7:**
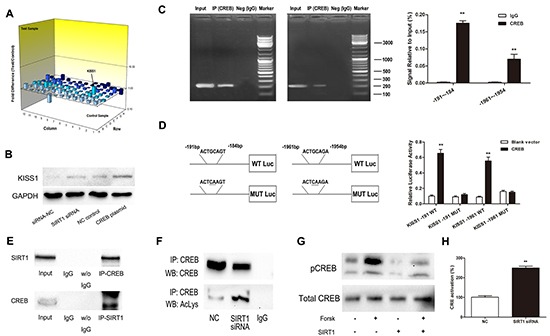
SIRT1 regulates KISS1 expression through deacetylation of transcription factor CREB **A.** Gene expression profile array analysis in SW620 cells. Three-dimension (3D) profile of the expression values for mRNA of siRNA-SIRT1 cells versus siRNA-NC cells. **B.** Upregulation of KISS1 protein was detected when either knockdown of SIRT1 or overexpression of CREB by Western blot. **C.** ChIP assays for CREB and its binding motif. Antibodies anti-IgG and anti-CREB were used in the ChIP assays. QRT-PCR was performed to quantify the binding activity. **D.** KISS1 promoter constructs containing a potential CREB binding motif (−191 to −184 bp and −1961 to −1954 bp). The transcriptional activity of the KISS1 promoter was greatly increased after co-expression of CREB and KISS1 reporter in wild type. **E.** Endogenous SIRT1 immunoprecipitates with CREB. Proteins immunoprecipitated with rabbit IgG was used as control. **F.** Downregulation of SIRT1 increases the immunoprecipitated CREB levels detected using anti-acetyl lysine antibody. **G.** Western blot showing that SIRT1 overexpression suppresses forskolin-induced CREB-Ser^133^ phosphorylation level. **H.** SIRT1 siRNA increases CRE transcriptional activity. Results are expressed as Luc/Renilla ratios. **P<0.01.

To elucidate the mechanism of influence of SIRT1 on KISS1 expression, we utilized JASPAR and TFSEARCH online software to predetermine the transcription factor of KISS1. CREB obtained the highest predicting score, and both prediction software programs pointed it, as one of the potential transcription factors of KISS1 promoter region. After transfection of CREB plasmid, KISS1 protein expression was significantly increased, as determined using Western blot (Figure 7B). For further analysis, ChIP and luciferase reporter assays were performed. The results not only indicated that CREB could actually bind to the promoter of KISS1 on −191~−184 bp and −1961~-1954 bp (Figure 7C), but also showed that the co-expression of CREB and KISS1 reporter resulted in an increase of KISS1 promoter activity (Figure 7D). These findings strongly testified that CREB was an activator of KISS1 expression.

### SIRT1 deacetylates CREB and regulates its activation

To identify the molecular mechanisms underlying the association between SIRT1 and CREB, we performed co-immunoprecipitation assay. As illustrated in Figure 7E-F, SIRT1 coimmunoprecipitated with CREB and SIRT1 siRNA increased the levels of acetylated CREB detected in the acetyl-lysine immunoprecipitates. Moreover, SIRT1 overexpression lowered the forskolin-induced pCREB-Ser^133^ phosphorylation level much more considerably than that of the negative control (Figure 7G) and inhibition of SIRT1 strongly up-regulated CRE transcriptional activity (Figure 7H).

## DISCUSSION

Distant metastasis, one of the crucial hallmarks of cancer, is the major cause of death in patients with cancers, especially in CRC. In spite of this negative evidence, there is a paucity of effective treatment options for managing patients with CRC metastasis. Thus, it is urgent to identify the factors involved in metastasis and understand the molecular mechanisms underlying metastatic CRC. At present, it is becoming largely accepted that miRNAs play important roles in cancer invasion and metastasis [24, 25]. Therefore, the identification of metastasis-related miRNAs and clarification of their functional mechanism may reveal the existence of novel predictors or therapeutic targets for the effective prevention or treatment of metastatic CRC patients.

In this study, we acquired both clinical and experimental evidence supporting the critical role of miR-199b in the suppression of SIRT1/CREB/KISS1 signaling pathway in the metastasis process of CRC. This is the first investigation to directly analyze miR-199b expression in primary CRC tissues and matching liver metastatic tissues and to show significantly downregulated expression of miR-199b in metastatic lesions. We demonstrated that the decreased miR-199b expression is correlated with poor prognosis in patients with CRC, and is closely associated with the late TNM stage and distant metastasis, whereas it is not related to tumor size, differentiation, and lymphatic invasion. In addition, multivariate analysis showed that the low miR-199b expression and distant metastasis were two independent prognostic factors for a poor overall survival rate of CRC patients. All of the above results suggest that miR-199b might be a novel prognostic predictor in CRC.

MiR-199b is now known to play a crucial role in carcinogenesis. For example, downregulation of miR-199b with increased mTOR was observed in endometrioid endometrial carcinoma [26]. Underexpressed miR-199b was found to target HIF-1oin hepatocellular carcinoma [27] and prostate cancer [28]. Moreover, Joshi et al. [29] discovered that miR-199b downregulation was closely associated with imatinib resistance in chronic myeloid leukemia. Recently, a growing body of evidence has indicated that miR-199b is involved in the process of metastasis in various types of cancers. Lauvrak et al. [30] reported that miR-199b-5p and miR-100-3p were downregulated in highly aggressive osteosarcoma cell lines. Other researchers found that miR-199b was associated with metastasis and prognosis in renal clear cell carcinoma [31]. However, the function of miR-199b in CRC and CRC metastasis is still not clarified. In the present study, we addressed this gap in knowledge. Functionally, we observed a weaker invasion and migration ability and elevated sensitivity of CRC cells to chemotherapeutic medicines after miR-199b overexpression. More importantly, we used an *in vivo* study to convincingly demonstrate that miR-199b significantly repressed distant metastasis of CRC cells. Thus, these clinical and experimental findings described above clearly and strongly support the conclusion that miR-199b is a suppressor of CRC metastasis. However, it is suggested that the context-dependent function of miR-199b in different types of cancer might be highly dependent on its targets of signaling pathways [31, 32].

SIRT1 has a large number of histone and non-histone substrates and deacetylates these proteins to regulate their functions. Our previous examinations have shown that SIRT1 overexpression was closely correlated with advanced- stage and poor prognosis in patients with CRC [23]. Chen et al. [33] further showed that SIRT1 deficiency attenuated the abilities of colony and sphere formation, and inhibited tumorigenicity of CRC cells. However, the mechanism of SIRT1 functions in CRC metastasis has yet to be fully elucidated. In the present investigation, we showed that miR-199b could significantly inhibit the expression of SIRT1 by directly combining to the 3′UTR of SIRT1 mRNA. Meanwhile, the suppression of miR-199b expression resulted in upregulation of SIRT1. In contrast, the knockdown of SIRT1 also led to appreciable effects on miR-199b expression, suggesting that there might be a loop regulation between miR-199b and SIRT1. Silencing of SIRT1 expression resulted in functional inhibition of invasion and migration of CRC cells, an effect was similar to that of miR-199b overexpression. Therefore, we concluded that the regulation of SIRT1 might be one of the mechanisms by which miR-199b exerts its metastasis suppressor functions.

To further explore the mechanisms responsible for SIRT1 dysregulation in the metastatic process of colorectal cancer, we employed array analysis of human tumor metastatic genes after silencing of SIRT1 in CRC cells. Our results indicated that the expression of KISS1 increased significantly when inhibition of SIRT1 occurred. KISS1 is one of the major metastasis suppressors that is inactivated in the majority of cancer types [34]. Recent studies revealed that KISS1 silencing increased cancer invasion and metastasis in breast cancer [35, 36] and bladder cancer [37] through driving progression of epithelial-mesenchymal transition (EMT). Furthermore, in CRC, KISS1 deficiency was found to predict poor prognosis and enhance the invasion and migration abilities of CRC cells [38, 39]. On the basis of these previous findings, we have reasons to suppose that SIRT1 regulates the metastasis of CRC through affecting KISS1 expression. However, the mechanism is still unknown. Hence, through bioinformatics assay we interestingly found that CREB was one of the potential transcription factors for KISS1 promoter. ChIP and luciferase reporter assays further verified that CREB could directly bind to −191 to −184 bp and −1961 to −1954 bp of KISS1 promoter to regulate KISS1 expression. Previous studies indicated that SIRT1 deacetylated CREB and suppressed its phosphorylation, leading to inactivation of CREB and then further affecting the downstream signaling pathways [40]. In our present study, we demonstrated that SIRT1 protein directly interacted with CREB protein and deacetylated CREB at the post-translational level in CRC. CREB deacetylation status led to suppression of the forskolin-induced CREB phosphorylation, which was in accordance with the conclusions outlined above.

In summary, our current investigation identified miR-199b as a miRNA type that is a significant suppressor of metastasis in CRC. We found that its expression level was frequently decreased in CRC metastases and was closely related to overall survival of CRC patients. Importantly, we revealed that SIRT1 was negatively regulated by miR-199b, causing a subsequent increase of the level of acetylated CREB, which in turn activates the KISS1 signaling pathway. Therefore, the investigation of the mechanism underlying the dysregulation of miR-199b in CRC might provide important clues to improve the understanding of CRC progression, contribute to the development of potential biomarkers for CRC prognosis, and guide the exploration of effective therapeutic targets for CRC.

## MATERIALS AND METHODS

### Patients and tissue samples

A total of 60 pathologically confirmed CRC patients were enrolled in the study and underwent surgery between 2010 and 2011. There were 16 cases with hepatic metastasis from which samples were collected and used for miRNA microarray assay. The specimens were obtained and immediately frozen in liquid nitrogen and stored at −80°C until RNA or protein extraction. All patients provided written informed consent before the samples were collected. The study was approved by the local Research Ethics Committee of Peking University People's Hospital, Beijing, China.

### MiRNA microarray analysis

Total RNA, containing miRNA, was extracted from tissue samples using TRIzol reagent (Invitrogen, Carlsbad, CA, USA) according to the manufacturer's instructions. The specific steps were conducted as previously described [41]. The expression levels of miRNAs in CRC tissues and CRC liver metastatic tissues were evaluated by Agilent human miRNA 8 × 15 k microarray (V16.0). Data were normalized and analyzed with Agilent Gene Spring software.

### Cell lines and cell culture

The human CRC cell lines SW480, SW620, HT29, LoVo, HCT116, and RKO cells were purchased from the American Type Culture Collection (Manassas, VA, USA). NCM460 cell line was purchased from American INCELL Corporation. Cells were maintained at 37°C with 5% CO_2_ in RPMI1640 medium (HT29, LoVo, HCT116, RKO) and Leibovitz's L-15 medium (SW480 and SW620) supplemented with 10% fetal bovine serum (FBS, Gibco), 100 U/mL penicillin (Sigma-Aldrich, St Louis, MO, USA), and 100 μg/mL streptomycin (Sigma-Aldrich).

### Quantitative real-time PCR

Total RNA extraction and quantitative real-time PCR (qPCR) were conducted according to the manufacture's instruction (Takara, Shiga, Japan), and data analysis was performed as we previously described [25]. The expression level of mRNAs or miRNAs was normalized with reference to GAPDH or U6 RNA, respectively. Primer sequences are shown in Supplementary Table S1.

### Transient transfection and stable infection

The SIRT1 interference sequence was obtained as described previously [16].A final concentration of 50 nM SIRT1 siRNA, miR-199b mimics, miR-199b inhibitor, or their corresponding negative controls (Ribobio Co., Guangzhou, China) were used for transient transfection with Lipofectamine 2000 (Invitrogen, Carlsbad, CA, USA). Lentiviral vectors (GeneChem Co. Ltd, Shanghai, China) containing miR-199b (LV-miR-199b) or negative control sequences were applied to infected CRC cells for conducting an animal study.

### Expression profiles of human tumor-metastasis-associated genes

The genomic expression level of total RNA from SW620 cells transfected with SIRT1 siRNA or NC was detected by RT^2^ Profiler™ PCR Array Human Tumor Metastasis (Qiagen China Co., Ltd., Shanghai, China). Fold-changes in gene expression were determined by the normalized signal intensities. Genes were supposed to have significant differential expression if they fit the criteria of *P*-values <0.05 and fold change > |1.5|.

### Western blot analysis

CRC cells were scraped and lysed in hypotonic lysis buffer (Thermo Fisher Scientific, Waltham, MA, USA). Equal amounts of protein were denatured in SDS sample buffer and separated in 10% or 15% polyacrylamide gels and then transferred to polyvinylidene difluoride (PVDF) membranes (Millipore, Hertfordshire, UK). The detailed procedures were performed as described previously [41]. The primary antibodies used in the experiments are shown in Supplementary Table S2.

### Tumor cell invasion and wound healing assay

For the cell invasion assay, SW480 and SW620 cells transfected with different reagents were placed into the upper chamber (24-well plates, 8-μm pore size, Corning) with the matrigel-pre-coated membrane. L-15 medium with 30% FBS was used as a chemoattractant. After incubation for 48 h, the invasive cells that had moved to bottom of the membrane were stained, counted, and photographed under a microscope (magnification ×200).

For the cell motility assay, CRC cells treated with mimics, siRNAs, or NC were seeded in 6-well plates to near confluence. A linear wound was generated by scratching the surface of the plates with a 20-μL sterile pipette tip. Then, the cells were washed with phosphate-buffered saline and incubated in 1% Leibovitz's L-15 medium. The width of wound gaps was photographed at 0 and 48 h. The results were assessed by measuring the gap width in multiple fields. This invasion and migration detection was repeated three times in duplicate.

### Cell chemosensitivity assay

The cell chemosensitivity was assessed by CCK8 assay. The chemotherapeutic drugs 5-FU (Sigma-Aldrich) and Oxaliplatin (Sigma-Aldrich) were used in this assay. The protocols were performed as previously described [42].

### Luciferase reporter assay

SW620 cells in 24-well plates were co-transfected with luciferase vectors (wild or mutant) for SIRT1 and either miR-199b mimics or NC mimics or were co-transfected with luciferase vectors containing the KISS1 promoter sequences with or without CREB luciferase reporter plasmids. For CRE gene reporter assay, pCRE-Luc plasmids were co-transfected with pTK-Renilla plasmids (Genomeditech, Shanghai, China). After 48 h, luciferase activity was measured by the Dual-Luciferase reporter assay system (Promega, Madison, WI, USA). Data are presented as ratios between firefly and renilla activity.

### Animal models

Four-week-old female athymic BALB/c nude mice were purchased from Beijing Vital River Laboratories (Beijing, China). These animals were maintained in a specific pathogen-free environment in the Experimental Animal Center of Peking University People's Hospital in accordance with the guidelines for Institutional and Animal Care and Use Committees.

For distant metastases assay, miR-199b-overexpression SW620 cells were injected into the caudal vein of mice. The mice were sacrificed on day 40 after cell inoculation or when the mice were moribund. The livers and lungs were then removed. Thesurface metastases were counted after dividing them into individual lobes. Every surface metastasis was examined by two independent investigators unaware of the experimental protocol and was scored separately [43]. The tissues were then placed in 10% buffered formalin, immersed in an ascending series of alcohols, and embedded in paraffin. Further, the specimens were cut into sections that were stained with hematoxylin and eosin.

### Immunoprecipitation analysis (IP)

Cells were lysed on ice in RIPA buffer (Beyotime, Jiangsu, China) along with adding protease inhibitor (Roche Diagnostics, Basel, Switzerland). After centrifugation, 50 μL of each sample were collected as input control. Each tube containing equal amounts of proteins was incubated with the respective specific antibody overnight at 4°C and was subsequently cultured with prewashed protein A+G sepharose beads (Beyotime). The beads were then washed three times with RIPA buffer. The protein-protein complexes were eluted from the beads by boiling and subjected to SDS-PAGE and Western blot.

### Chromatin immunoprecipitation (ChIP)

ChIP assay was performed using SW620 cells, and the specific steps were followed according to Yamaguchi's instructions [44]. Briefly, cells were consecutively cross-linked by incubation in 1% formaldehyde-containing medium for 10 min at room temperature, sonicated to an average fragment size of 500 bp, and centrifuged to remove cell debris. A small portion (10%) of each sample was removed as an input control, and anti-CREB (9197#, Cell Signal Technology) and the rabbit normal IgG antibody (2729#, Cell Signal Technology) were added to the remaining samples. The protein–DNA complex was collected with protein A Sepharose beads (Millipore), and then eluted and reverse cross-linked. DNA was recovered using a PCR purification kit (Qiagen, Hilden, Germany) and assessed by real-time PCR.

### Statistical analysis

Unless otherwise explained specifically, all results were expressed as mean ± SEM and analyzed using the SPSS 20.0 software (SPSS, Chicago, IL, USA). Differences between groups were assessed using the Student's *t*-test. The relationship between miR-199b expression and the clinicopathological features of CRC was analyzed by the Pearson's χ2 test. The relationship between the expression of miR-199b and SIRT1 was evaluated by Spearman's correlation. The overall survival of the two patients groups was analyzed by the log-rank test using the Kaplan-Meier method. *P* < 0.05 was considered statistically significant.

## SUPPLEMENTARY FIGURES AND TABLES


