# Supplementary material for: Downregulation of miR-199b is associated with distant metastasis in colorectal cancer via activation of SIRT1 and inhibition of CREB/KISS1 signaling
Source: Oncotarget. 2016 Apr 27;7(23):35092–105. doi: 10.18632/oncotarget.9042 (PMC5085212; doi:10.18632/oncotarget.9042)
Supplement: Supplementary file 1 [file oncotarget-07-35092-s001.pdf]

SUPPLEMENTARY FIGURES AND TABLES

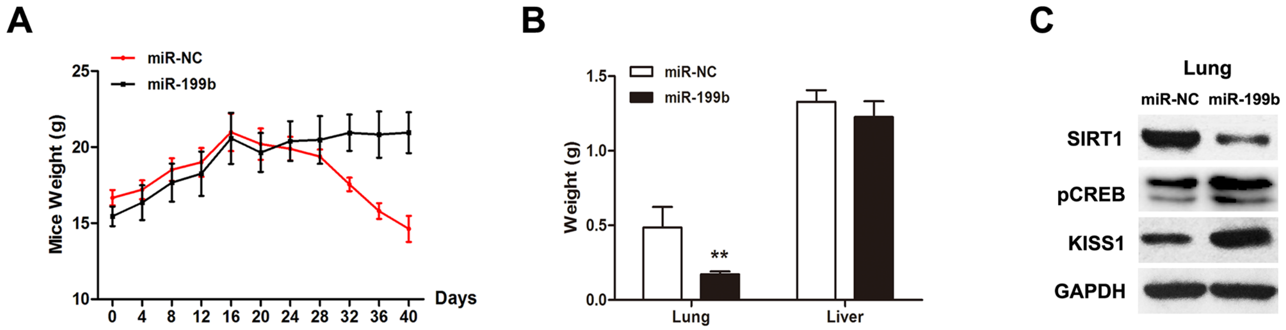

Supplementary Figure S1: Upregulation of miR-199b results in suppression of metastasis of SW620 cells *in vivo*. A. Mice weight of each group during the mice grown process. NC group showed weight loss after 16 days injection. **B.** Weight of lungs and livers were measured from the metastasis model. The weight of NC group seemed heavier than that of miR-199b overexpression group. **C.** SIRT1, pCREB and KISS1 expression level were measured using Western Blot in tissues from lungs.**P<0.01.

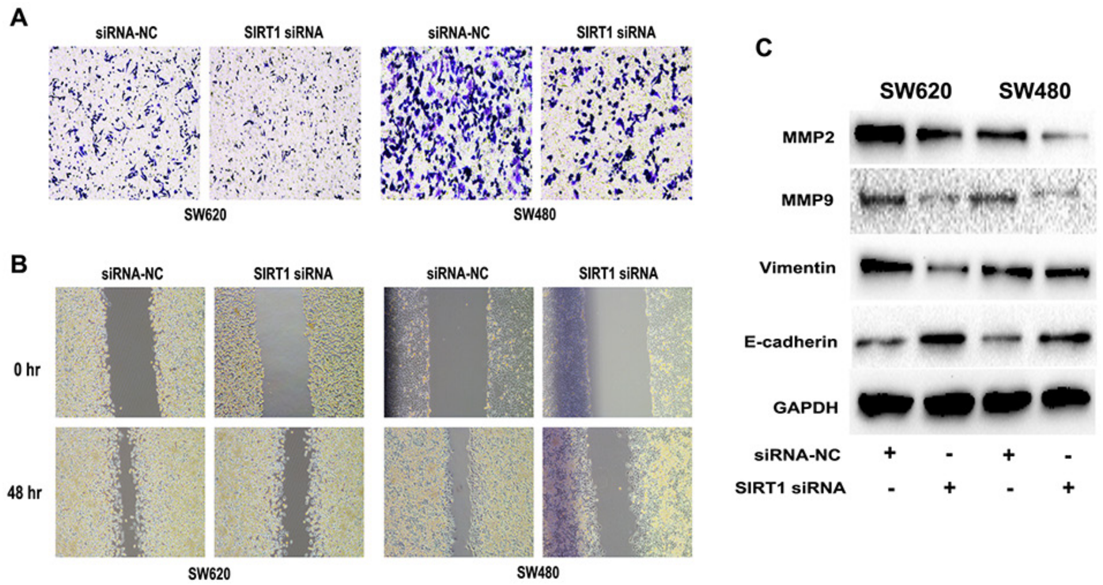

Supplementary Figure S2: Knockdown of SIRT1 inhibits cell invasion and migration in SW620 and SW480 cell lines. A. Inhibition of SIRT1 expression repressed cell invasion of SW620 and SW480 cells. **B.** Silencing of SIRT1 led to weakening migration ability of CRC cells. **C.** Western blot analysis assessed protein level changes of MMP2, MMP9, Vimentin and E-cadherin after treated with siRNA-SIRT1.

Supplementary Table S1: Primer sequences of genes

Gene	Primer sequences
miR-199b	Forward: 5’-CCCAGTGTTTAGACTATCTGTTC-3’
U6	Forward: 5’-CTCGCTTCGGCAGCACA-3’
SIRT1	Forward: 5’-AAATGCTGGCCTAATAGAGTGG-3’<br>Reverse: 5’-TGGCAAAAACAGATACTGATTACC-3’
GAPDH	Forward: 5’-CCCCGGTTTCTATAAATTGAGC-3’<br>Reverse: 5’-CACCTTCCCCATGGTGTCT-3’
KISS1 (ChIP)	Forward: 5’-AGAGGGTGTGGAGGATGGAA-3’<br>Reverse: 5’-AGCTCCCTGATCACATCCCT-3’<br>Forward: 5’-ACATATCCGGACCTCCCCAA-3’<br>Reverse: 5’-CTGAGCCTTAGACACACGCA-3’

Supplementary Table S2: Western Blot primary antibodies.

Antibody	Corporation	Dilution ratio
Anti-GAPDH (#2118)	Cell Signal Technology	1:1000
Anti-SIRT1 (sc-15404)	SantaCruz Biotechnology	1:1000
Anti-CREB (#9197)	Cell Signal Technology	1:1000
Anti-p-CREB (#9198)	Cell Signal Technology	1:1000
Anti-MMP2 (#4022)	Cell Signal Technology	1:500
Anti-MMP9 (#13667)	Cell Signal Technology	1:500
Anti-E-cadherin (#3195)	Cell Signal Technology	1:1000
Anti-Vimentin (#5741)	Cell Signal Technology	1:1000
Anti-acetyl lysine (#9441)	Cell Signal Technology	1:500
Anti-KISS1 (sc-15400)	SantaCruz Biotechnology	1:200

Supplementary Table S3: Gene expression profiles array after treated with siRNA-SIRT1 in SW620 cells

Gene name	Fold change	Expressed after knockdown of SIRT1
**KISS1**	**2.24705**	**Up**
TRPM1	1.857705	Up
IGF1	1.827481	Up
CXCR4	1.713929	Up
CST7	1.707649	Up
SRC	1.697056	Up
NR4A3	1.594991	Up
NF2	1.570819	Up
ETV4	1.555651	Up
EWSR1	1.536158	Up
TCF20	1.480172	Up
FN1	1.471329	Up
MYCL	1.466075	Up
CCL7	1.463801	Up
GAPDH	1.458766	Up
IL1B	1.427771	Up
SMAD2	1.415902	Up
CTSK	1.407068	Up
CXCR2	1.397773	Up
MDM2	1.363847	Up
MGAT5	1.358312	Up
CTNNA1	1.357375	Up
MMP3	1.352322	Up
EPHB2	1.348355	Up
TP53	1.331378	Up
APC	1.320607	Up
CD44	1.309848	Up
METAP2	1.305436	Up
SMAD4	1.301232	Up
MTA1	1.29948	Up
SYK	1.28477	Up
DENR	1.256562	Up
CD82	1.247505	Up
MET	1.245641	Up
CDKN2A	1.241566	Up

(*Continued*)

Gene name	Fold change	Expressed after knockdown of SIRT1
BRMS1	1.239961	Up
MMP11	1.236387	Up
RORB	1.236003	Up
CTSL	1.230665	Up
CTBP1	1.227442	Up
IL18	1.225459	Up
FLT4	1.22079	Up
TIMP4	1.212688	Up
VEGFA	1.210234	Up
SERPINE1	1.206345	Up
MMP9	1.205592	Up
NME1	1.200743	Up
ITGB3	1.194326	Up
MCAM	1.192628	Up
HRAS	1.190056	Up
GNRH1	1.189919	Up
TIMP3	1.187937	Up
RPSA	1.187678	Up
PLAUR	1.185667	Up
HPSE	1.185449	Up
TGFB1	1.180046	Up
CXCL12	1.179634	Up
HGF	1.179634	Up
MMP13	1.171764	Up
HPRT1	1.16882	Up
CDH1	1.165596	Up
FAT1	1.149481	Up
CHD4	1.146746	Up
PTEN	1.138921	Up
KISS1R	1.135941	Up
RPLP0	1.134961	Up
HTATIP2	1.133161	Up
ITGA7	1.130504	Up
SSTR2	1.112709	Up
NME4	1.111201	Up
MMP2	1.106438	Up

(*Continued*)

Gene name	Fold change	Expressed after knockdown of SIRT1
PNN	1.102698	Up
FXYD5	1.094984	Up
TIMP2	1.077983	Up
MYC	1.068855	Up
TNFSF10	1.067955	Up
KRAS	1.056906	Up
COL4A2	1.053885	Up
SET	1.037131	Up
MTSS1	1.018297	Up
MMP7	1.01596	Up
RB1	-1.01693	Down
CDH11	-1.02187	Down
B2M	-1.06535	Down
TSHR	-1.14061	Down
FGFR4	-1.16499	Down
MMP10	-1.29633	Down
ACTB	-1.81644	Down
CDH6	-2.38742	Down
